# Decreased Autocrine EGFR Signaling in Metastatic Breast Cancer Cells Inhibits Tumor Growth in Bone and Mammary Fat Pad

**DOI:** 10.1371/journal.pone.0030255

**Published:** 2012-01-19

**Authors:** Nicole K. Nickerson, Khalid S. Mohammad, Jennifer L. Gilmore, Erin Crismore, Angela Bruzzaniti, Theresa A. Guise, John Foley

**Affiliations:** 1 Medical Sciences Program, Indiana University School of Medicine, Bloomington, Indiana, United States of America; 2 Division of Endocrinology, Department of Medicine, Indiana University School of Medicine, Indianapolis, Indiana, United States of America; 3 Indiana University Cancer Center, Indiana University School of Medicine, Indianapolis, Indiana, United States of America; 4 Department of Oral Biology, Indiana University School of Dentistry, Indianapolis, Indiana, United States of America; 5 Department of Anatomy and Cell Biology, Indiana University School of Medicine, Indianapolis, Indiana, United States of America; 6 Department of Dermatology, Indiana University School of Medicine, Indianapolis, Indiana, United States of America; University of Medicine and Dentistry of New Jersey, United States of America

## Abstract

Breast cancer metastasis to bone triggers a vicious cycle of tumor growth linked to osteolysis. Breast cancer cells and osteoblasts express the epidermal growth factor receptor (EGFR) and produce ErbB family ligands, suggesting participation of these growth factors in autocrine and paracrine signaling within the bone microenvironment. EGFR ligand expression was profiled in the bone metastatic MDA-MB-231 cells (MDA-231), and agonist-induced signaling was examined in both breast cancer and osteoblast-like cells. Both paracrine and autocrine EGFR signaling were inhibited with a neutralizing amphiregulin antibody, PAR34, whereas shRNA to the EGFR was used to specifically block autocrine signaling in MDA-231 cells. The impact of these was evaluated with proliferation, migration and gene expression assays. Breast cancer metastasis to bone was modeled in female athymic nude mice with intratibial inoculation of MDA-231 cells, and cancer cell-bone marrow co-cultures. EGFR knockdown, but not PAR34 treatment, decreased osteoclasts formed *in vitro* (p<0.01), reduced osteolytic lesion tumor volume (p<0.01), increased survivorship *in vivo* (p<0.001), and resulted in decreased MDA-231 growth in the fat pad (p<0.01). Fat pad shEGFR-MDA-231 tumors produced in nude mice had increased necrotic areas and decreased CD31-positive vasculature. shEGFR-MDA-231 cells also produced decreased levels of the proangiogenic molecules macrophage colony stimulating factor-1 (MCSF-1) and matrix metalloproteinase 9 (MMP9), both of which were decreased by EGFR inhibitors in a panel of EGFR-positive breast cancer cells. Thus, inhibiting autocrine EGFR signaling in breast cancer cells may provide a means for reducing paracrine factor production that facilitates microenvironment support in the bone and mammary gland.

## Introduction

The epidermal growth factor (EGFR) has long been recognized as a therapeutic target in breast and other epithelial cancers due to its ability to potently stimulate cell proliferation, motility, and invasion. The EGFR is activated by a family of ligands that include epidermal growth factor (EGF), Amphiregulin (AREG), transforming growth factor α (TGFα), heparin-binding EGF (HB-EGF), betacellulin, epiregulin, epigen, and Neuregulin 2β [1]. These factors are synthesized as plasma membrane proteins tethered by a transmembrane domain, requiring proteolytic cleavage to be accessible to receptors [2]. These individual ligands may induce differential signaling pathways downstream of the EGFR, both from the plasma membrane and intracellular compartments, which can result in certain ligands being more efficient stimulators of proliferation [1], [3], [4], [5], [6]. Breast cancer cells frequently express the EGFR, one or more of its ligands and proteases that shed the ligands, resulting in autocrine signaling that may contribute to their rapid growth and invasive behavior.

The EGFR is frequently expressed in the basal subtype of breast cancer, which typically lack the expression of estrogen receptor α (ERα), progesterone receptor (PR) and Her2 receptor, accounting for only ∼15–20% of the total disease [7], [8], [9]. However, 50–75% of basal breast cancers express EGFR and are more aggressive than similar tumors lacking the receptor [10], [11]. Co-expression of the ADAM17 protease and the TGFα ligand in primary basal tumors has been associated with reduced survival [12]. These observations suggest that more aggressive basal-like breast cancers have the capacity to be stimulated by autocrine EGFR signaling, whereas the ligands produced by other subtypes of breast cancer (luminal, HER2 positive) may serve as paracrine signaling molecules [13].

Models of breast cancer metastasis to specific organs have provided evidence that EGFR ligands mediate paracrine signaling with cells of the tumor environment. Recent gene expression profiling of a bone-homing MDA-231 subline found that MMP-1 (matrix metalloproteinase 1) and ADAMTS-1 (a disintegrin and metalloproteinase with thrombospondin motifs) were upregulated, leading to increased AREG shedding [14]. The increased AREG appeared to signal via the EGFR present on osteoblasts, leading to reduced production of osteoprotegrin, the decoy ligand to the major controller of osteoclast differentiation and activation, receptor for nuclear factor κβ ligand (RANKL) [14]. Increased osteoclast numbers and activity is a key element in the growth of breast cancer cells in the bone [15]. The metastatic growth of these MDA-231 sublines could be inhibited by the EGFR-targeted therapeutics cetuximab or gefitinib alone, or in combination with other targeted agents [14], [16], [17].

Autocrine activation of EGFR on breast cancer cells may also influence signaling with the bone microenvironment. Models of bone metastasis have provided evidence that cancer cell activation of EGFR often leads to the production of paracrine signaling molecules necessary for survival and rapid growth within the bone. Among the most well characterized factors that facilitate the growth of cancer cells in the bone is parathyroid hormone related protein (PTHrP), which signals through its receptor on osteoblasts, and leads to an increase of RANKL expression and increased osteoclast activity [18], [19]. Autocrine activation of EGFR is a major regulator of PTHrP in both breast and lung cancers [20]. Intriguingly, the stimulation of the PTH receptor on osteoblasts stimulates the expression and shedding of AREG, thus potentially initiating a second autocrine loop of EGFR signaling in osteoblasts [21], [22]. Taken together, autocrine EGFR-driven cytokine production, as well as paracrine interactions of the EGFR ligands themselves, both appear to drive growth of bone-metastatic lesions suggesting various agents that disrupt this signaling could be effective treatments for breast cancer metastasis to bone.

In this study, we evaluated EGFR ligand expression by a bone-homing subline of the human breast cancer cell line MDA-231, with regard to their impact on specific malignant phenotypes and breast cancer cell signaling, as well as paracrine signaling to a mouse bone cell line. To specifically inhibit autocrine signaling in the MDA-231 cells we reduced EGFR expression by a lentiviral shRNA, and to inhibit both autocrine and paracrine EGFR signaling, an AREG neutralizing antibody was used. Finally, we evaluated the impact of altered autocrine and paracrine signaling on MDA-231 cell growth *in vitro* as well as *in vivo*, in the bone and mammary fat pad.

## Materials and Methods

### Ethics Statement

Animal care and experiments were approved by the Indiana University Animal Care and Use Committee (IACUC), OLAW assurance #94094-01, protocol #10-014.

### Cell lines and cell culture

MDA-MB-231 cells were obtained from T. Guise [18] and MC3T3 cells were obtained from A. Robling [23], and were grown in DMEM (Sigma, St Louis, MO) supplemented with 10% FBS (Atlanta Biologicals, Lawrenceville, GA) and 10 ng/mL insulin (Sigma). S1T3 and NS2T2A1 cells were both obtained from Z. Bouizar [24], and grown in a 50∶50 mixture of RPMI and DMEM:F12 (Sigma) supplemented with 10% FBS. SUM149 cells were purchased from Asterand (Detroit, MI) and grown in F12 Hams (Sigma) supplemented with 10% FBS (Atlanta Biologicals).

Production of shEGFR-MDA-231 and shControl cells: MDA-MB-231 cells [18] were plated in 12-well dishes and grown to 50% confluence. 10 µL of either EGFR shRNA lentiviral particles (Santa Cruz Biotechnology, Santa Cruz, CA) or Control shRNA lentiviral particles (Santa Cruz Biotechnology) with 6 µg/mL polybrene (Santa Cruz Biotechnology) was added to the wells. Cells were grown for 24 hours before removal of lentiviral particles, then grown another 24 hours before 1.5 µg/mL puromycin selection. Pooled colonies were tested for EGFR expression by western blotting, and cultures maintained in media with 1. 5 µg/mL puromycin.

### Animal injections and therapeutic dosing

For intratibial inoculation, either 7.5×10^3^ MDA-MB-231, shEGFR-MDA-231, or shControl cells were inoculated into the left tibia of 3–4 week old female athymic nude mice (Harlan, Indianapolis, IN). Mice were anesthetized with 5% isoflurane and laid in a dorsal position. Autoclaved 100 µL Hamilton syringes with 27 gauge needles were used to puncture the skin at the proximal end of the left tibia. The syringe was gently pushed through the epiphysis to about 3 mm deep to assure it passed the through metaphysis, and 10 µL of cell suspension was inoculated slowly over 20 seconds. Mice were anesthetized with 5% Isoflurane, and lay in a prone position for weekly radiography to monitor lesion progression (35 kV for 10 seconds; Faxitron, Lincolnshire, IL). Animals were x-rayed at 2× magnification, and lesions were first detected at 14 days post inoculation. End point for these studies was 4–6 weeks after tumor inoculation, or earlier if the size of the x-ray lesion reached 25% of the upper tibia area, swelling of knee region exceeded 2-fold the diameter of the non-injected limb, the limb could not be used for ambulation, or the animals displayed signs of excessive pain, per our veterinarian-guided animal protocol and pain-scale. Osteolytic area on x-ray was measured using ImageJ software (NIH).

Upon sacrifice, hind limbs were removed and fixed in 10% neutral buffered formalin for 48 hours, and then 70% EtOH for at least 24 hours. After microCt imaging, bones were decalcified (10% EDTA for 1 week), and embedded in paraffin. Tibiae were sectioned at 7 µM in the sagittal plane and mid-sagittal sections were stained with hematoxylin and eosin (H&E) or tartrate resistant acid phosphatase (TRAP). TRAP staining for osteoclasts was performed using an azo-dye coupling method with fast red violet LB salt (F-3881, Sigma) as described [25]. After rehydration through graded alcohols, sections were incubated in freshly prepared TRAP stain at 37°C for 15 minutes, counterstained in hematoxylin, and mounted in glycerin jelly. Serial slides were stained with hematoxylin and eosin (H&E) as described [26]. For this study, PAR34 antibody was administered for 4 weeks through intraperitoneal injection once weekly at 10 mg/kg in a sterile 0.9% saline solution [27].

For mammary fat pad tumors, shControl or shEGFR-MDA-231 cells were combined with 50% Matrigel and inoculated at 1×10^6^ cells in 100 µl total volume in the first mammary fat pad of female athymic nude mice, aged 3–4 weeks (Harlan). One group of shControl mice was administered PAR34 at 10 mg/kg/week by intraperitoneal injection. Tumors were measured twice weekly for length (L) and width (W), and tumor volume (V) calculated as: V = (L×W^2^)×0.5.

### Micro-CT

Fixed tibiae were scanned using a SkyScan micro-CT (SkyScan 1172; SkyScan, Belgium) as previously described [28], with the following scanner settings: voltage, 60 kV; resolution, 6 µm; 0.5 mm aluminum filter; stage rotation, 0.7; and frame-averaging, 2. Flat-field corrections were used to minimize background noise. NRecon software (SkyScan), was used to reconstruct the images, with post-alignment optimization performed for each separate tibia. CTan software (SkyScan), was used to analyze reconstructed images, separating bone from surrounding soft tissue with a threshold range of 100 to 255 (binarized 0–255 scale). Bone volume was reported from analysis of 700 sections per tibia. 3D images were obtained using MeshLab software (MeshLab, 3D-CoForm) with smoothing option.

Trabecular bone analysis regions were chosen in the secondary spongiosum, with a consistent total length of 1 mm measured for each tibia. Region of interest was chosen as only the internal bone cavity containing trabecular bone with cortical bone excluded.

### Osteoclastogenesis assays

Osteoclastogenesis assays were performed as in [29]. Briefly, 1–4 month old mice were euthanized, hind limbs dipped in 70% ethanol and removed at hip. Femur and tibia ends were cut to expose the bone marrow cavity, and each marrow cavity flushed with 5–10 mL of DMEM cell culture medium. 50 µL of cell suspension was mixed with 450 µL of 2% acetic acid to lyse red blood cells, and remaining cells counted. 4×10^5^ cells were plated in each well of a 24-well dish with 60 ng/mL RANKL (PeproTech) for 3 days. After 3 days, 2×10^3^ MDA-231, shControl, or shEGFR-MDA-231 cells were plated with the bone marrow cells, with 60 ng/mL RANKL and 10 ng/mL MCSF (PeproTech) and grown for 3 days before TRAP staining. For TRAP staining, cells were washed with 1×PBS, fixed with ice cold methanol for 10 minutes, and stained in fresh TRAP solution for 15 minutes at 37°C. TRAP solution was replaced with 1×PBS for cell counting under the microscope.

### Statistical analysis

Results of *in vitro* experiments are expressed as the mean ± SD of triplicate or quadruplicate measures of independent replicates for single experiments. Results of *in vivo* experiments are expressed as the mean ± SEM of three to six replicates of samples taken from ten individual animals. All statistical comparisons were based on two-tailed analysis of the Student's *t* test. A *P* value of <0.05 was considered to be significant.

## Results

### Amphiregulin is highly secreted by MDA-231 cells

**Figure 1:**
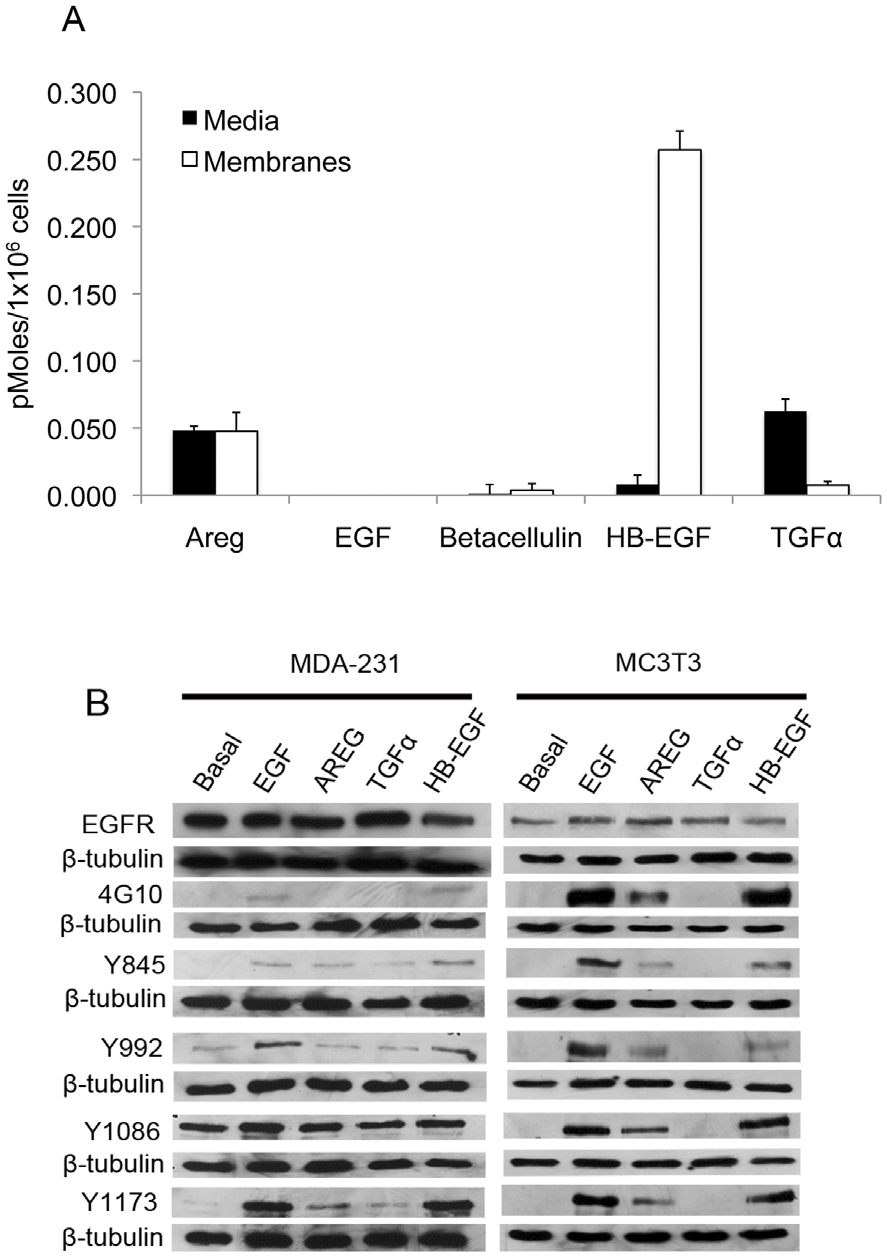
EGFR ligand expression and shedding in MDA-231 cells. (A) ELISA measurement of media or membrane extracts from MDA-231 cells. Measurements were taken from two independent cultures and performed in triplicate. (B) Western blots of anti-EGFR and anti-phosphorylated tyrosine resides in MDA-231 or MC3T3 cells treated with EGF, AREG, TGFα, or HB-EGF.

Previously we determined a subline of the aggressive breast cancer cell line MDA-231 efficiently colonizes mouse bone after intracardiac inoculation, expresses high levels of EGFR protein and modest levels of the ErbB2 and ErbB3 receptors, and sheds AREG [20]. To more completely evaluate EGFR ligand production in these cells, we examined the expression of five EGFR ligands, including epidermal growth factor (EGF), AREG, betacellulin, heparin-binding EGF (HB-EGF), and transforming growth factor α (TGFα) using ELISA for both conditioned media and membrane extracts. MDA-231 cells release high levels of AREG (0.048pM), and maintain similar concentrations associated with the membrane fraction. Surprisingly, higher levels of HB-EGF also remained associated with the cell membrane (0.254pM), with lower levels (0.008pM) detectable in the media (Fig. 1A). Low concentrations of TGFα were present on the membrane fraction (0.008pM) while higher levels were detected in the media (0.063pM). Betacellulin was present in low concentrations (0.004pM) and EGF protein was undetectable using this methodology (Fig. 1A). In terms of autocrine signaling *in vitro*, AREG appears to be shed at the highest concentrations, while high levels of membrane-associated HB-EGF indicate that this could be the predominant ligand if it were cleaved from the membrane.

### Amphiregulin activates EGFR phosphorylation on both MDA-231 and MC3T3 cells

To determine if the impact of EGFR signaling in the bone microenvironment is similar to that of breast cancer cells, we used a mouse preosteoblastic cell line MC3T3 as a model to compare receptor phosphorylation induced by exogenous ligand treatment. Here, we used 100 nM recombinant human ligands (AREG, TGFα, and HB-EGF), as well as recombinant human EGF (10 nM) to serve as the prototype ligand. MC3T3 or MDA-231 cells were treated with EGF, AREG, TGFα, or HB-EGF, followed by western blotting with their respective phospho-specific antibodies. Modest levels of basal EGFR tyrsosine phosphorylation could be detected in MDA-231 cells at Y992 and Y1086, whereas baseline EGFR levels could not be detected in the MC3T3 line with any of the phospho-specific antibodies (Fig. 1B). Human EGF and HB-EGF were able to induce receptor phosphorylation on both MDA-231 and MC3T3 cells, as detected with 4G10, a pan phosphotyrosine antibody, as well as the other site-specific antibodies. Exogenous AREG induced modest phosphorylation of some residues in MDA-231 cells compared to EGF, but appeared to increase phosphorylation of all tested residues in MC3T3 cells. We noted that TGFα caused very little phosphorylation in the human cells and was not able to induce detectable changes in EGFR phosphorylation in mouse MC3T3 cells. Though both AREG and TGFα are shed and capable of inducing receptor phosphorylation in MDA-231 cells, AREG appears to be the highest cleaved ligand and it is able to potently activate the EGFR on mouse osteoblast-like MC3T3 cells providing the rationale to target this ligand as the main inducer of both autocrine breast cancer signaling and paracrine receptor signaling in mouse tissues.

### shRNA to the EGFR causes a decrease in migration and PTHrP expression in MDA-231 cells

**Figure 2:**
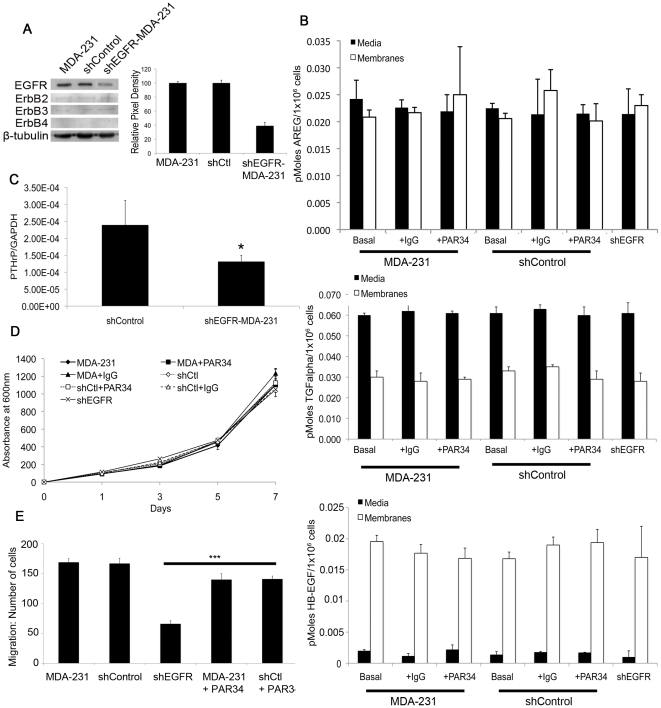
Characterization of the shEGFR-MDA-231 cell line. (A) Extracts from MDA-231, shControl, and shEGFR-MDA-231 cells, probed with anti-EGFR or anti-ErbB2, ErbB3, or ErbB4 antibodies and anti-β-Tubulin (loading control). Histogram notes relative pixel density of EGFR protein of shEGFR-MDA-231 cells versus shControl and MDA-231 cells. (B) AREG, TGFα, and HB-EGF ELISA measurements of MDA-231, shControl, and shEGFR-MDA-231 cells, to verify no changes in basal or PAR34 treated ligand expression. ELISA measurements were performed in triplicate from two separate cultures. (C) Relative PTHrP mRNA levels in the shControl and shEGFR-MDA-231 cell lines. PTHrP was measured by qRT-PCR analysis and relative ratios of PTHrP mRNA to GAPDH mRNA levels were shown (mean of triplicate measures from a single experiment; bars, SD). (D) MTT proliferation assays were performed on shEGFR-MDA-231, MDA-231, and shControl cells, as well as PAR34-treated MDA-231 or shControl cells. MTT measurements were performed in quadruplicate, p<0.05. (E) 24 hour migration assay of shEGFR-MDA-231, MDA-231, and shControl cells, with PAR34 treatment to the latter two lines, p<0.001. Migrated cells were obtained from two separate migration wells, with four random fields chosen for counts from each well.

To inhibit breast cancer cell autocrine and paracrine signaling, we used shRNA to the EGFR as well as a monoclonal antibody (PAR34) (Figure S1). To reduce autocrine EGFR signaling in the MDA-231 line, cells were transduced with a lentiviral shRNA to the receptor (shEGFR-MDA-231 cells) or a shRNA scrambled control (shControl). As detected by western blot, there was a 64% knockdown of the EGFR as compared to MDA-231 or shControl cells, and this knockdown not affect levels of other EGFR family receptors (Fig. 2A). Introduction of the shEGFR construct had no effect on production of AREG, TGFα, or HB-EGF mRNA production (data not shown). We verified by ELISA that ligand protein levels were not disrupted by the shEGFR construct, as AREG, TGFα, and HB-EGF were present in the media or on cell membranes at the same levels in shEGFR-MDA-231 cells, as compared to MDA-231 and shControl cells (Fig. 2B). Treatment of MDA-231 or shControl cells with PAR34 antibody or control IgG had no effect on ligand expression of AREG, TGFα, or HB-EGF (Fig. 2B). As expected, a decrease (p<0.05) in PTHrP levels in the shEGFR-MDA-231 cells was observed as compared to control cells (Fig. 2C), indicative of reduced autocrine EGFR signaling.

We then examined impact of PAR34 on breast cancer cells grown *in vitro*. PAR34 inhibited exogenous AREG-induced phosphorylation of tyrosines 992 and 1173 in MDA-231 cells, when compared to IgG control (Figure S2B), and this inhibition was AREG-specific, as PAR34 did not inhibit stimulation by EGF. Similar inhibition of exogenous phosphorylation was noted in the non-tumorigenic, epithelial breast cell line S1T3 (S1 cells).

To further test the impact of autocrine EGFR signaling inhibition by PAR34 antibody and shRNA knockown, cell proliferation and migration were examined *in vitro*. EGFR signaling has been reported to stimulate motility, but does not induce proliferation in MDA-231 cells [30], [31]. Using the MTT assay we found that shEGFR-MDA-231 cells and controls treated with PAR34 proliferated at a similar rate to non-treated controls (Fig. 2C). As shown in Figure 2D, PAR34 inhibited migration (p<0.001) of both MDA-231 and shControl cells by 20%, and migration was decreased (p<0.001) by 65% in shEGFR-MDA-231 cells relative to controls. Taken together, these *in vitro* assays confirm that inhibition of EGFR by PAR34 or shRNA decreases breast cancer cell motility.

### PAR34 treatment modifies the trabecular patterning factor of bone

To examine the impact of inhibiting AREG signaling within the bone, we first evaluated PAR34 antibody treatment in non-tumor bearing animals. Female athymic nude mice aged 3–4 weeks received intraperitoneal injections of PAR34, at 10 mg/kg/week, for 4 weeks. Upon sacrifice, tibiae were removed and prepared for microCT and histological sectioning. While PAR34 treatment did not affect the gross bone structure, as analyzed by both x-ray and microCT (data not shown), microCT showed a decrease (p<0.001) in trabecular pattern factor in PAR34 tibiae when compared to vehicle treated animals (Table S1 and Figure S2A). We also evaluated osteoclasts present in the newly deposited bone under the hypertrophic zone of growth plate chondrocytes, and observed an increase (p<0.01) in the number of these cells per bone surface area in PAR34 treated animals versus control animals (Figure S2B and Table S1). Overall, PAR34 treatment influenced bone growth, thus validating this dose and schedule as effective for targeting bone *in vivo*.

### shEGFR-MDA-231 cells produce smaller tumors in bone

**Figure 3:**
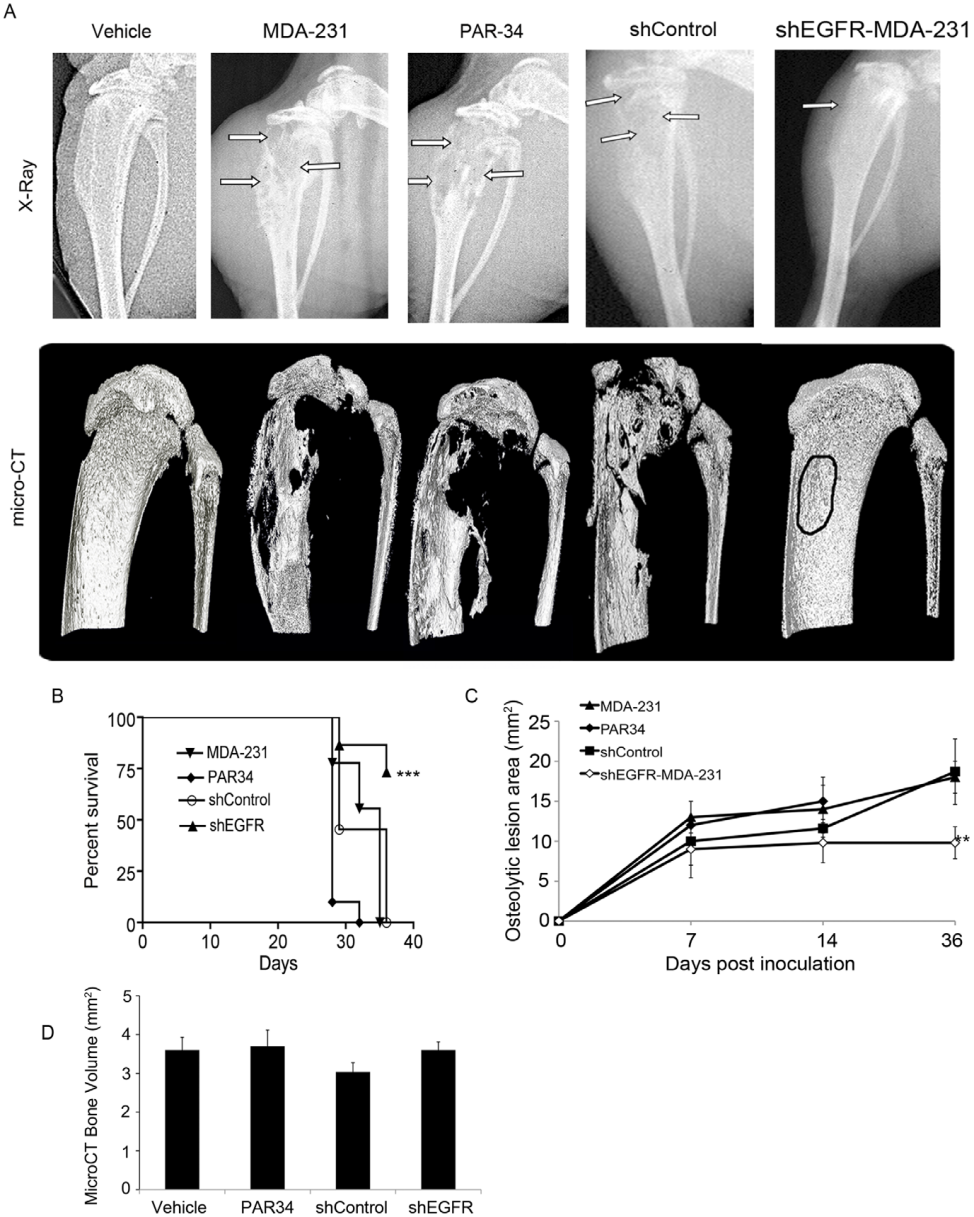
In vivo analysis of autocrine or paracrine inhibition of EGFR. (A) Representative end point x-rays for each treatment group (top row), with arrows denoting osteolytic lesion areas. Corresponding 3D micro-CT images (bottom row). n = 10 animals per treatment group. (B) Kaplan-meyer survival curve demonstrating significant increased survival in the shEGFR-MDA-231 injected animals, p<0.001. n = 10 animals per group. (C) Osteolytic lesion area was measured using ImageJ software from x-ray images. n = 10 mice, p<0.01. PAR34-treated animals required sacrifice at the 3-week time point due to maximum allowable lesion areas and pain scale (per our animal protocol). (D) Micro-CT bone volume analysis of tibiae in all treatment groups. 700 sections were analyzed per tibia. p = not significant.

To examine global inhibition of AREG signaling, or to specifically reduce cancer cell EGFR signaling during osteolytic lesion growth within the bone, female athymic nude mice (aged 3–4 weeks) were inoculated in the left tibia with MDA-231, shControl, or shEGFR-MDA-231 cells. Intratibial inoculation was chosen to insure that differential motility of the shEGFR-MDA-231 did not inhibit colonization of the bone. Three days after inoculation, treatment of one group of MDA-231 inoculated mice was initiated with weekly intraperitoneal injection of PAR-34 antibody (10 mg/kg). The MDA-231, shControl, and PAR34 treated groups had extensive osteolytic lesion destruction as detected by x-ray and microCT at the experimental end-point, while the majority of shEGFR-MDA-231 mice had smaller regions of distinct bone loss measured by x-ray (Fig. 3A). All PAR34 treated animals required sacrifice after the 3-week time point, as they displayed experimental end-point criterion including maximum x-ray lesion size, swelling of the injected limb, or ambulation difficulties. Survival was increased (p<0.001) in shEGFR-MDA-231 tumor-bearing mice as compared to those inoculated with MDA-231 or shControl (Fig. 3B), and osteolytic lesion size was decreased (p<0.01) in shEGFR-MDA-231 animals (Fig. 3C). Although large lesions were readily apparent in the reconstruction of microCT scans from the MDA-231, shControl, or PAR34 groups, total tibia head bone volume was not significantly different as compared to the shEGFR-MDA-231 group (Fig. 3D).

**Figure 4:**
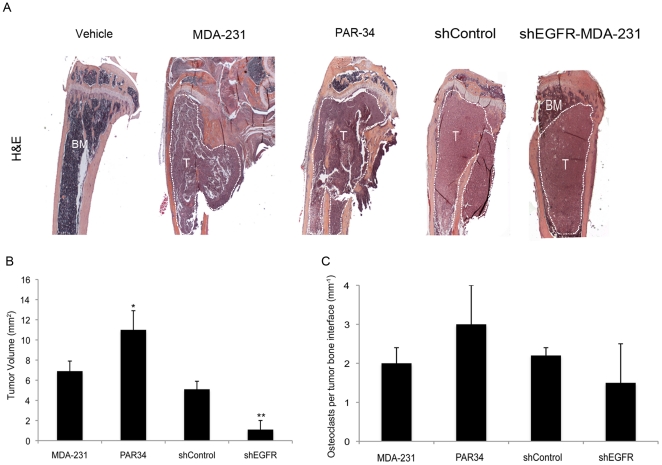
Histomorphometric analysis of tumor bearing bones. (A) Representative images of H&E stained tibiae from each treatment group. Tumor region outlined in white, BM = bone marrow, T = Tumor. (B) Histomorphometric tumor volume analysis on H&E stained tibia sections. Care was taken to measure the same size tissue volume on each section. *p<0.05 and **p<0.01. (C) Osteoclast counts of TRAP stained slides from each treatment group. p = not significant, n = 10 mice per group.

Examination of H&E stained tibiae from all groups verified large, destructive tumors within the MDA-231, PAR34 treated, and shControl groups (Materials and Methods S1). Interestingly, shEGFR-MDA-231 animals had smaller tumors (p<0.01) that remained within the bone marrow cavity (Fig. 4A). Surprisingly, the PAR34 treated animals had a larger tumor volume (p<0.05) when compared to controls (Fig. 4B). Tartrate resistant acid phosphatase (TRAP) staining indicated the number of osteoclasts per tumor bone interface in shEGFR-MDA-231 bones trended toward a decrease in comparison to MDA-231 or shControl tumor-bearing tibiae (Fig. 4C). Additionally, we observed an increase in osteoclasts per tumor bone interface, though this was not significant (Fig. 4C).

Thus, it appears that decreased EGFR was sufficient to reduce the size of osteolytic lesions and tumor volume within bone. Conversely, PAR34 antibody enhanced MDA-231 growth within the bone.

### Modulation of EGFR signaling impacts osteoclastogenesis in vitro

**Figure 5:**
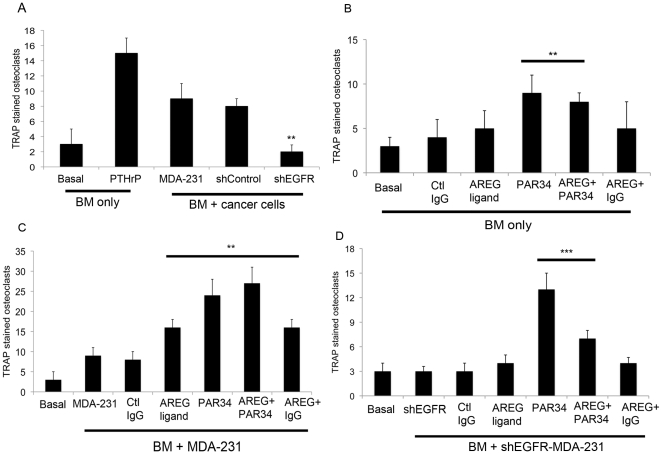
Activated osteoclast measurement by bone marrow and cancer cell co-culture. (A) Co-cultures of mouse bone marrow with MDA-231, shControl, or shEGFR-MDA-231 cells were TRAP stained to identify active osteoclasts. Four random fields were counted from two separate wells for each co-culture. **p<0.01. (B–D) Co-cultures of mouse bone marrow with MDA-231 cells, (C) bone marrow only, or (D) bone marrow with shEGFR-MDA-231 cells were treated with AREG ligand, PAR34 antibody, Control IgG antibody, or a combination of ligand with antibody as noted. Wells were TRAP stained to identify active osteoclasts, and four random fields were counted form two separate wells for each treatment. **p<0.01, ***p<0.001. (E) bone marrow only or co-cultured with MDA-231 cells were treated with 1 µM gefitinib or DMSO control for 3 days followed by TRAP staining for active osteoclasts. ***p<0.001.

We also examined the effects of EGFR knockdown or PAR34 treatment using an *in vitro* osteoclastogenesis assay, whereby MDA-231 or shEGFR-MDA-231 cells were co-cultured with mouse bone marrow (BM) to determine if osteoclast formation could be increased. As seen in Figure 5A, co-culture of BM with shEGFR-MDA-231 cells stimulated fewer (p<0.01) osteoclasts than control cell co-cultures, correlating with the decreased osteolytic lesion size *in vivo.*

Next we evaluated the impact of PAR-34 and exogenous AREG on various permutations of the co-culture assay. We also observed that PAR34 antibody caused an increase in osteoclasts in BM alone (Fig. 5B, p<0.01) or co-cultures with MDA-231 (p<0.01) and the shEGFR-MDA-231 cells (p<0.001) (Fig. 5C&5D). In contrast, exogenous AREG ligand failed to increase osteoclasts in BM, but stimulated the formation in co-cultures that contained MDA-231 cells (Fig. 5C, p<0.01). Intriguingly exogenous ligand did not increase osteoclast number in the shEGFR-MDA-231 containing co-cultures (Fig. 5D). The impact of PAR-34 on BM alone or cancer cell co-cultures generally corresponded with *in vivo* findings where the antibody treatment produced increased osteoclasts in non-tumor bearing bones and increased tumor size in cancer cell injected bones.

To further investigate the impact of EGFR signaling inhibitors on osteoclastogenesis, BM and MDA-231 co-cultures were treated with a range of concentrations of gefitinib, a small molecule EGFR inhibitor [32]. As shown in Figure S3, 1 µM gefitinib also increased osteoclasts (p<0.001) in co-cultures, but showed a trend toward decreased formation in BM cultures alone. These findings coupled with those from PAR-34 treatments suggest that different EGFR inhibitors can have distinct impacts on osteoclastogenesis and in some cases they may enhance it.

### shEGFR-MDA-231 cells produce smaller mammary fat pad tumors

**Figure 6:**
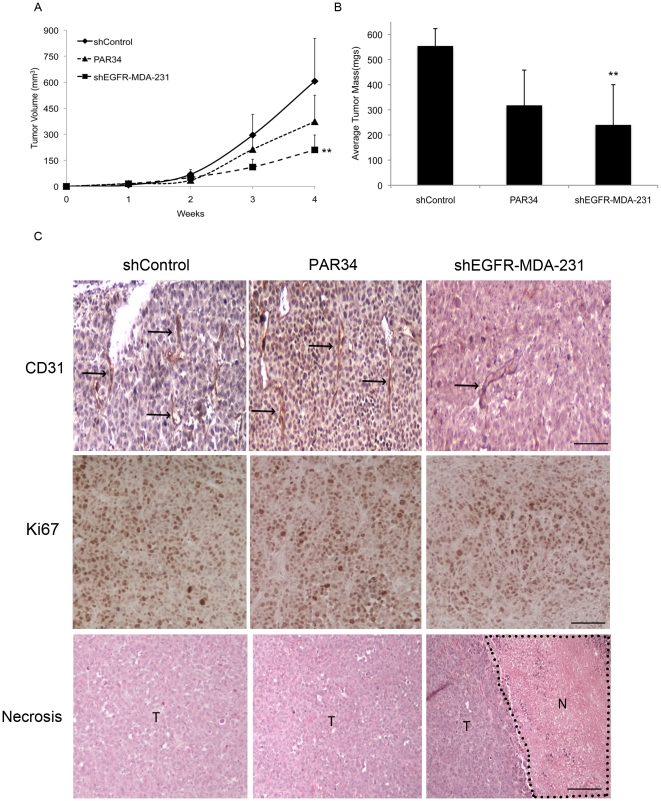
shEGFR-MDA-231 cells produce smaller tumors in the mammary fat pad. (A) Tumor volume measurement for mammary fat pad tumors grown from injection of shControl, PAR34 treated shControl cells, or shEGFR-MDA-231 cells. PAR34 treated animals were administered 10 mg/kg/week of PAR34 by intraperitoneal injection. Tumor measurements were taken three times per week. **p<0.01, n = 6 mice per group. (B) Upon sacrifice, tumor masses were assessed. **p<0.01, n = 6 mice per group. (C) Paraffin-embedded tumors were stained with anti-CD31 antibody for vessel formation (top row), black arrows denote areas of vessel staining. Ki67 staining (middle row) was examined for cellular proliferation. shEGFR-MDA-231 tumors contained large regions of necrosis, as seen in Necrosis in the bottom row. T = tumor, N = necrotic region. No necrosis was observed in shControl or PAR34 treated tumors. Vessel and proliferation counts, as well as percent changes of necrotic regions are noted in Table 1. n = 6 animals per treatment group. Magnification bars, CD31 and Ki67 = 100 µm. Necrosis = 1 mm.

To determine if the reduced growth of the shEGFR-MDA-231 cells in bone was specific to that microenvironment, we examined the *in vivo* growth rate of mammary fat pad tumors produced by shControl or shEGFR-MDA-231 cells. A group of shControl-inoculated animals were treated with weekly intraperitoneal injections of PAR34 (10 mg/kg). As shown in Figure 6, tumor volume measures and final masses were decreased (p<0.01) in shEGFR-MDA-231 tumors as compared to shControl (Fig. 6A&6B). While PAR34 treatment trended towards reduced tumor volume and mass, these results were not significant compared to shControl tumors (Fig. 6A&6B). Histological analysis revealed an increased (p<0.05) necrotic area in shEGFR-MDA-231 tumors, despite unchanged cell proliferation as detected by an anti-Ki67 antibody (Table 1). However, fewer vessels (p<0.001) were stained by anti-CD31 antibody in the shEGFR-MDA-231 tumors than shControl cells (Table 1 and Fig. 6C). Thus, reduced growth *in vivo* of the shEGFR-MDA-231 cells was observed in the mammary fat pad, likely correlated with reduced vascularization of the tumor.

**Table 1:** Histological Tumor Analysis.

	Necrosis		Ki67		CD31	
	% Necrotic Tumor Area (mm^2^)	Fold Increase	Number Cells	Fold Increase	Mean Vessel Count	% Reduction
**MDA-231**	16	N/A	31±6	N/A	22±3	N/A
**PAR34**	19	1.2	34±7	1.09	20±2	9.1
**shEGFR-MDA-231**	29	1.9*	32±11	1.03	7±2	68.2***

### Decreased EGFR signaling causes a reduction in proangiogenic factor expression

**Figure 7:**
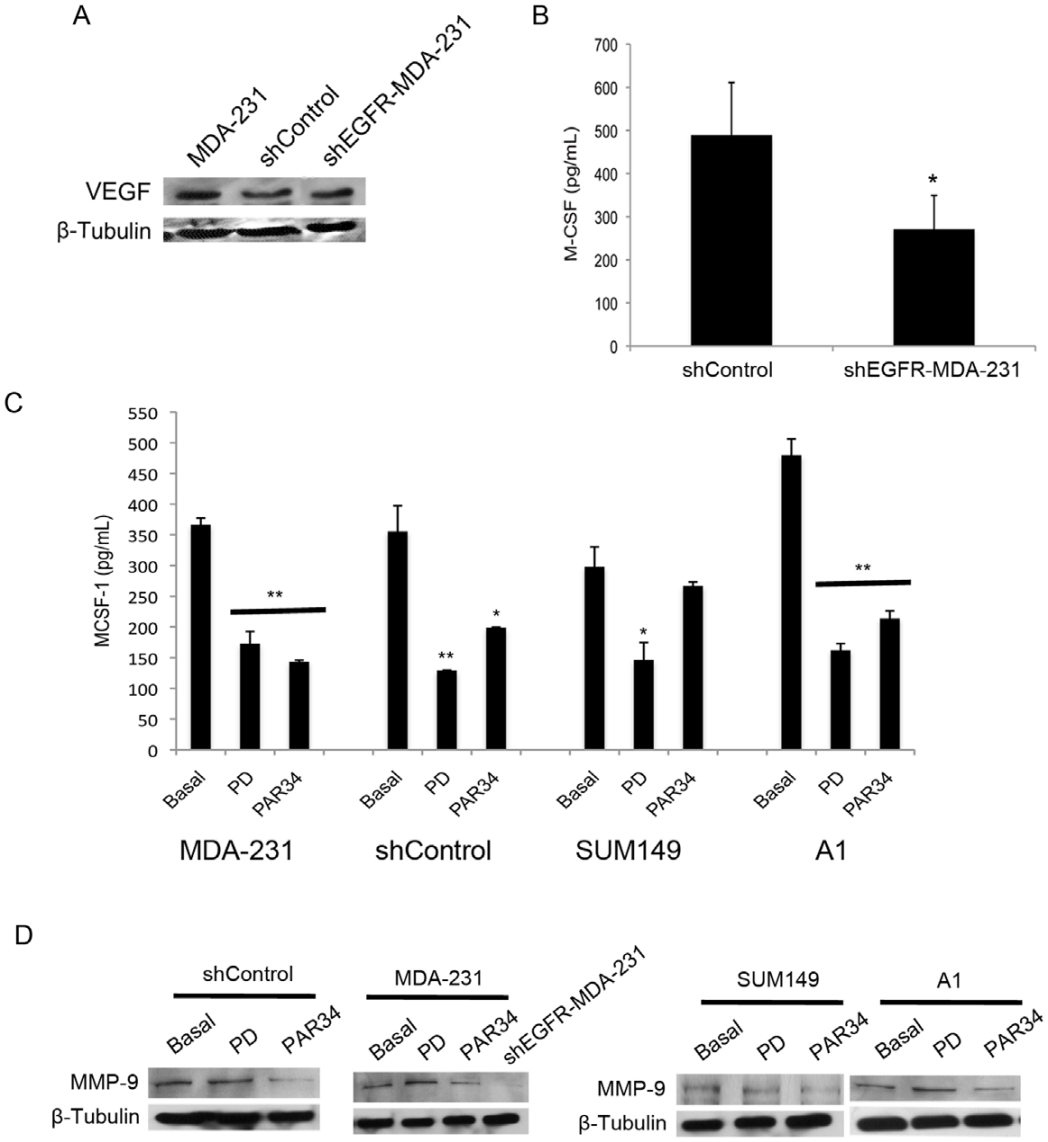
MCSF-1 and MMP-9 decrease with EGFR inhibition. (A) anti-VEGF probed western blot for MDA-231, shControl, and shEGFR-MDA-231 extracts, β-tubulin used for loading control. (B) Media was harvested from shControl or shEGFR-MDA-231 cells and analyzed for MCSF-1 by ELISA, *p<0.05. Measurements were obtained from two separate cultures, and performed in triplicate. (C) MDA-231, shControl, SUM149, or NS2TA1 cells were treated with the tyrosine kinase inhibitor PD153035 (10 µg/mL) compound for 6 hours or PAR34 (10 µg/mL) for 24 hours before media harvest for MCSF-1 ELISA, *p<0.05 and **p<0.01. Measurements were obtained from two separate cultures, and performed in triplicate. (D) anti-MMP9 antibody probed western blots for shControl, MDA-231, SUM149, or NS2TA1 cell extracts treated with PD153035 (10 µg/mL) compound for 6 hours or PAR34 (10 µg/mL) for 24 hours. shEGFR-MDA-231 cells were untreated. anti-β-tubulin used as loading control.

To explore the molecular basis of the reduced vasculature of the mammary fat pad tumors produced by shEGFR-MDA-231 cells, we first examined changes in expression of vascular endothelial growth factor (VEGF). Previous work has reported VEGF is regulated in breast cancer cells by EGFR signaling [33], however we observed no differences in control versus shEGFR-MDA-231 cells (Fig. 7A). Previous publications have noted that EGFR signaling regulates macrophage colony-stimulating factor-1 (MCSF-1) expression in murine cancer cells [34]. MCSF-1 could influence angiogenesis by recruiting macrophages or various progenitors from the bone marrow, which could produce VEGF or directly contribute to neoangiogenesis [35], [36]. MCSF-1 levels were lower (p<0.05) in shEGFR-MDA-231 cells when compared to controls, as measured by ELISA (Fig. 7B).

To verify this finding is due to EGFR inhibition and not off target effects of the shRNA construct, we examined MCSF-1 levels in a panel of breast cancer cell lines following treatment with the small molecule EGFR inhibitor PD153035 or PAR34. PD153035 reduced MCSF-1 secretion from the parental MDA-231 (p<0.01), shControl (p<0.01), SUM149 (p<0.05), and the tumorigenic epithelial breast cancer cell line NS2T2A1 (p<0.01) (Fig. 7C). PAR34 decreased MCSF-1 levels in MDA-231, shControl and NS2TA1 cells (p<0.05) in which AREG is the predominant ligand, but not in SUM149 cells. We further evaluated the shEGFR-MDA-231 and control cells for matrix metalloproteinase 9 (MMP-9), a protease that promotes angiogenesis by releasing VEGF that is bound to extracellular matrix [37]. As shown in Figure 7D, MMP-9 levels were markedly reduced in cell extracts of shEGFR-MDA-231, as compared to shControl and MDA-231. Also, PAR34 reduced expression of the protease in SUM149 and NS2TA1 cell lines. PD153035 inhibition (6-hrs) had no effect on MMP-9 levels. These findings suggest that autocrine EGFR signaling regulates at least two proangiogenic factors in breast cancer cell lines, and disruption of receptor signaling would be predicted to reduce vascularization and decreased growth of tumors.

## Discussion

In this study, we found that reduced EGFR expression decreased MDA-231 cell growth within bone and the mammary gland. Previous studies suggested that EGFR signaling promotes growth *in vivo* as part of paracrine relationships between breast epithelia-derived cells and the microenvironment. The mammary epithelium expresses both EGFR and its ligands EGF, TGFα, and AREG, suggesting a potential for autocrine signaling [38], [39]; however, elegant recombination experiments established that mammary gland ductal outgrowth requires EGFR expression in the fat pad, and AREG expression in the epithelium [39], [40], [41]. In lung and brain metastasis models, epigen or HB-EGF expressed by MDA-231 cells signal to the EFGR on endothelial cells to facilitate colonization of these organs. In a model of bone metastasis, breast cancer cell derived AREG is thought to signal to the osteoblast, facilitating osteoclast formation and driving osteolytic destruction. In contrast, autocrine EGFR signaling is typically associated with proliferation of epithelial cancers [13]. Since the MDA-231 line bears a mutation in K-ras, which activates the MAPK cascade the major driver of mitogenesis downstream of the EGFR [30], [31], this line represented an ideal system for modulating receptor levels without reducing cell proliferation. The shEGFR-MDA-231 cells did not exhibit alterations in the expression of ligands or other ErbB receptors, and had identical rates of proliferation as compared to control lines (Fig. 2). Decreased EGFR expression in the MDA-231 resulted in slower tumor growth in both the bone and the mammary gland (Figs. 3 and 6). The shEGFR-MDA-231cells produced smaller osteolytic lesions *in vivo* and induced the formation of fewer osteoclasts *in vitro* relative to controls (Figs. 3 and 5). Consistent with a central role for the breast cancer cell EGFR in the stimulation of osteoclastogenesis, the addition of exogenous AREG to co-cultures containing shEGFR-MDA-231cell failed to induce increased numbers of the bone resorbing cells (Fig. 5). We conclude that autocrine EGFR signaling contributes to MDA-231 tumor growth in bone and the mammary gland independent of driving cancer cell proliferation.

There is a growing appreciation that EGFR signaling in epithelial cancer cells stimulates the expression of many chemokines, cytokines, growth factors, and receptors that facilitate paracrine interactions with non-cancer cells of the tumor microenvironment [20], [42], [43], [44]. EGFR signaling controls the expression of VEGF isoforms, which are pivotal factors that control angiogenesis in many tumors [33], [42], [45]. We did not detect differences in VEGF levels in the shEGFR-MDA-231 cells, consistent with previous studies of MDA-231 sublines [46]. However, we did detect decreases in proangiogenic factors such as MCSF-1 and MMP-9 in the shEGFR-MDA-231 cells, as well as a panel of breast cancer cell lines treated with an EGFR tyrosine kinase inhibitor or PAR-34. Reduced expression of MCSF-1 and MMP-9 would likely influence the growth of the MDA-231 cells in both the mammary fat pad and the bone. Also, the decreased production of PTHrP would be expected to contribute to reduced growth of the MDA-231 line in bone. Previously, it has been shown that PTHrP antibody inhibition dramatically decreased the number of osteoclasts per tumor bone interface, coupled with decreased osteolytic lesion size [18]. Consistent with the reduction of PTHrP, we observed a trend in reduction of osteoclasts in shEGFR-MDA-231 inoculated tibiae; also, *in vitro* studies indicated that shEGFR-MDA-231 cells generate fewer osteoclasts than control MDA-231 cells (Fig. 5A). It is likely that the EGFR on breast cancer cells controls the expression of many additional cytokines and growth factors that mediate tumor cell-microenvironment interactions, both in primary tumors and sites of metastasis.

Our attempt to block both autocrine and paracrine EGFR signaling by antagonizing AREG interaction with its receptor, using PAR34 antibody, produced surprising results. Given that we had previously found that AREG was the major ligand controlling PTHrP expression in MDA-231 cells [20], it was not surprising that PAR-34 decreased MCSF-1 and MMP-9 production. However, the antibody only modestly inhibited MDA-231 cell motility in comparison to knockdown of the receptor (Fig. 2D). This raises questions as to whether the various ligands might exhibit differential impacts on cell motility and growth factor production, as previously established for some of these agonists in the stimulation of EGFR-dependent cellular proliferation [3]. Also, the failure of the antibody to potently inhibit motility may reflect its inability of block signaling of the EGFR from internal compartments such as the endosome [47]. *In vivo*, we observed that PAR-34 treatment increased MDA-231 tumor growth within the bone, while also increasing active osteoclasts at the tumor bone interface. Correspondingly, PAR34 increased osteoclastogenesis in BM alone as well as BM cancer cell co-cultures, and increased osteoclast numbers below the growth plate of non-tumor bearing. These later findings suggest that PAR-34 may induce a higher baseline bone turnover, and this could contribute to the increased tumor growth that we observed in our *in vivo* experiments. Although an impact on baseline osteoclastogenesis of BM cultures was not observed with gefitinb, we found that this EGFR inhibitor also increased osteoclastogenesis MDA-231 containing co-cultures. Together these unanticipated findings lead us to speculate that AREG-EGFR signaling in the bone marrow microenvironment may influence other processes besides osteoblast differentiation and subsequent osteoclastogenesis. Recent reports indicate that EGFR signaling decreases hematopoietic stem cell mobilization in response to G-CSF [48]. Derivatives of hematopoietic stem cells include osteoclasts, monocytes, myeloid suppressor cells and megakaryocytes that all could influence the growth of breast cancer cells in the bone [49]. Our unexpected findings with AREG antibody treatments of cancer cells in the bone marrow encourage a more careful analysis of the impact of various inhibitors on EGFR signaling on all cell types in the breast cancer bone metastasis microenvironment.

In conclusion, EGFR knockdown in MDA-231 cells reduced their motility and production of secreted factors that stimulate osteolytic lesion growth and angiogenesis *in vitro*. *In vivo*, EGFR knockdown in MDA-231 cells reduced tumor growth both in the mammary fat pad and the bone. MDA-231 cells act as a model for triple negative breast cancers, so these findings raise the possibility that interventions that could reduce EGFR expression in triple-negative breast cancer cells might provide therapeutic benefit to patients with metastatic disease.

## Supporting Information

Figure S1 **Model of inhibition of autocrine and paracrine EGFR signaling within the bone environment.** (A) In an unhibited situation, cancer cells produce and cleave AREG to act in autocrine or paracrine signaling. Autocrine EGFR signaling can activate the expression of paracrine factors such as PTHrP that can directly stimulate the PTH receptor on osteoblasts and this increases RANKL production and osteoclastogenesis. In addition, stimulation of the PTH receptor induces AREG-EGFR signaling on the osteoblast, leading to increased RANKL accessibility and oteoclastogenesis. Finally cancer cell derived AREG can stimulate the EGFR on the osteoblast in a paracrine manner resulting in increased RANKL accessibility and oteoclastogenesis (B) shEGFR knockdown in cancer cells will decrease autocrine signaling and AREG-EGFR signaling in the endosome, in turn decreasing PTHrP levels. Decreased PTHrP secretion will lead to decreased osteoblast RANKL production, and a decrease in osteolysis. However this should not prevent cancer cell derived AREG from stimulating the EGFR on osteoblasts. (C) PAR34 inhibition of AREG binding the EGFR on cancer cells will decrease PTHrP secretion, and thus decrease RANKL production by the osteoblast. PAR34 may also inhibit cancer cell and autocrine AREG from stimulating the osteoblast EGFR thus reducing RANKL accessibility and osteolysis.
(TIF)

Figure S2 **PAR34 inhibition on bone environment.** (A) Female athymic nude mice aged 3–4 weeks were treated with weekly intraperitoneal injection of PAR34 antibody at 10 mg/kg or an equal volume of sterile 0.9% saline as vehicle. Left column, parraffin-embedded tibiae were TRAP stained for active osteoclasts. Active osteoclasts were counted in the primary spongiosum directly under the growth plate. Arrows denote positively stained osteoclasts. Right column, microCT images were reconstructed from the secondary spongiosum, and denote changes in trabecular bone. For both TRAP staining and microCT analysis, n = 10 mice per group. Magnification bar = 170 µm. (B) MDA-231 or S1 cells were treated with AREG ligand with or without PAR34 antibody, and compared to PAR34 inhibition with EGF ligand treatment. Cell lysates were resolved on 8% SDS-PAGE gels before membrane transfer and probed with the corresponding tyrosine phosphorylated antibodies.
(TIF)

Figure S3 **Gefitinib treatment of bone marrow co-cultures.** Mouse bone marrow (BM) was cultured alone or co-cultured with MDA-231 cells followed by treatment with 0.5 µM, 1.0 µM, or 5 µM of EGFR kinase inhibitor gefitinib for three days. Osteoclasts were counted after TRAP staining from three random fields from two separate wells. *** p<0.001. & denotes all cultured cells in wells were dead after 5 µM gefitinib treatment.
(TIF)

Table S1 **MicroCT and histomorphometry measurements of PAR34 treated, non-tumor bearing tibiae.**
(TIF)

Methods S1 **Supporting Materials and Methods.**
(DOC)
