# Supplementary figures and images for: Decreased Autocrine EGFR Signaling in Metastatic Breast Cancer Cells Inhibits Tumor Growth in Bone and Mammary Fat Pad
Source: PLoS One. 2012 Jan 19;7(1):e30255. doi: 10.1371/journal.pone.0030255 (PMC3261896; doi:10.1371/journal.pone.0030255)

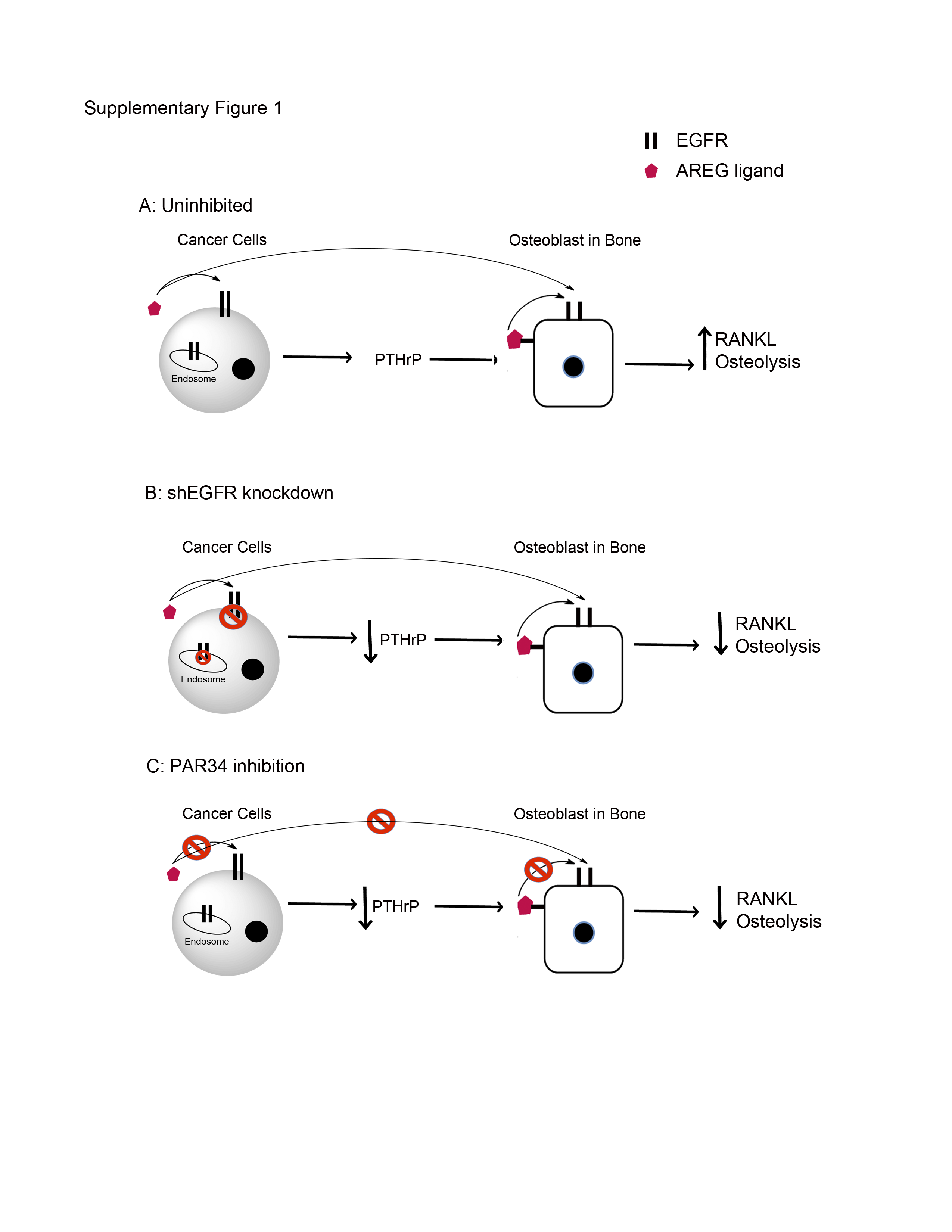

Supplement: Figure S1 — Model of inhibition of autocrine and paracrine EGFR signaling within the bone environment. (A) In an unhibited situation, cancer cells produce and cleave AREG to act in autocrine or paracrine signaling. Autocrine EGFR signaling can activate the expression of paracrine factors such as PTHrP that can directly stimulate the PTH receptor on osteoblasts and this increases RANKL production and osteoclastogenesis. In addition, stimulation of the PTH receptor induces AREG-EGFR signaling on the osteoblast, leading to increased RANKL accessibility and oteoclastogenesis. Finally cancer cell derived AREG can stimulate the EGFR on the osteoblast in a paracrine manner resulting in increased RANKL accessibility and oteoclastogenesis (B) shEGFR knockdown in cancer cells will decrease autocrine signaling and AREG-EGFR signaling in the endosome, in turn decreasing PTHrP levels. Decreased PTHrP secretion will lead to decreased osteoblast RANKL production, and a decrease in osteolysis. However this should not prevent cancer cell derived AREG from stimulating the EGFR on osteoblasts. (C) PAR34 inhibition of AREG binding the EGFR on cancer cells will decrease PTHrP secretion, and thus decrease RANKL production by the osteoblast. PAR34 may also inhibit cancer cell and autocrine AREG from stimulating the osteoblast EGFR thus reducing RANKL accessibility and osteolysis. (TIF) [file pone.0030255.s001.tif]

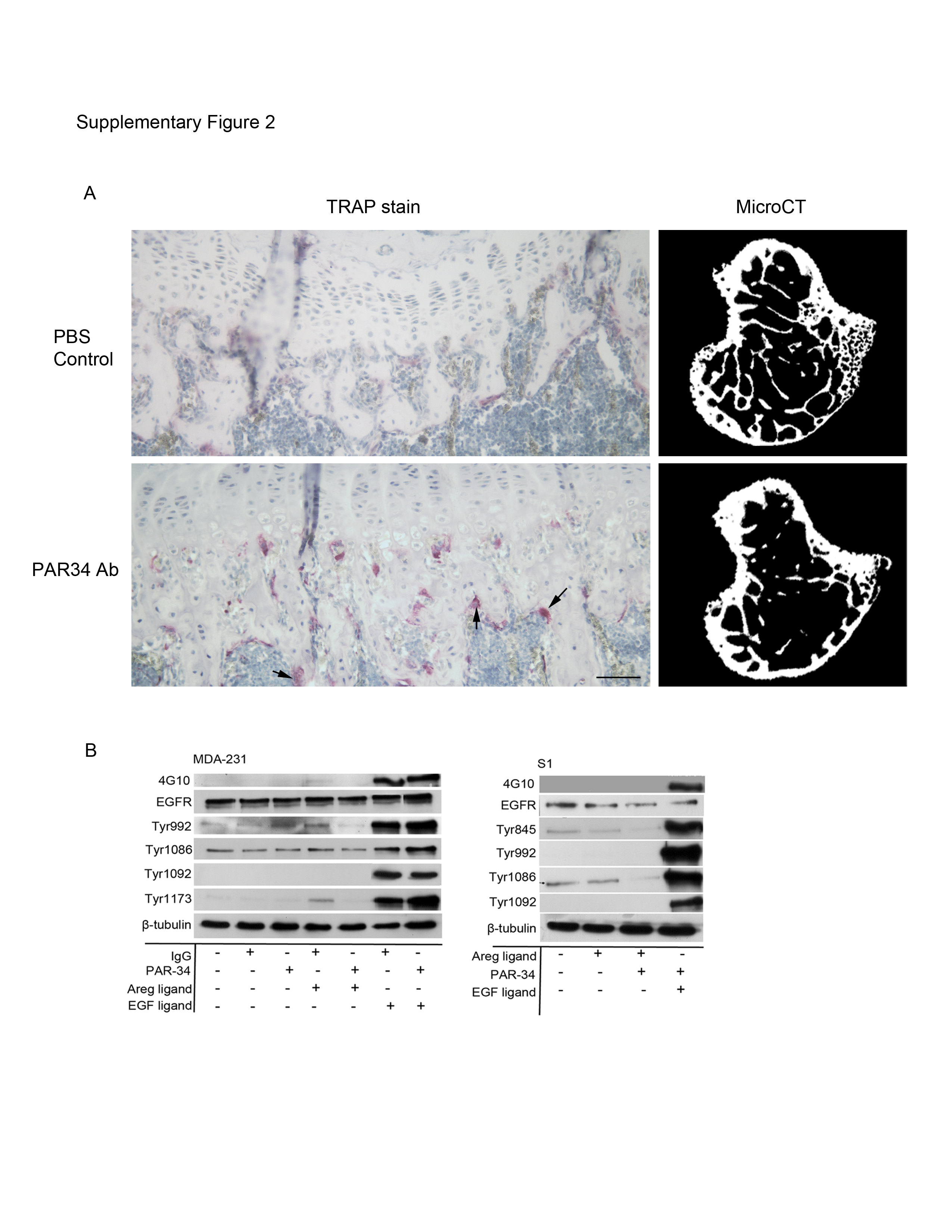

Supplement: Figure S2 — PAR34 inhibition on bone environment. (A) Female athymic nude mice aged 3–4 weeks were treated with weekly intraperitoneal injection of PAR34 antibody at 10 mg/kg or an equal volume of sterile 0.9% saline as vehicle. Left column, parraffin-embedded tibiae were TRAP stained for active osteoclasts. Active osteoclasts were counted in the primary spongiosum directly under the growth plate. Arrows denote positively stained osteoclasts. Right column, microCT images were reconstructed from the secondary spongiosum, and denote changes in trabecular bone. For both TRAP staining and microCT analysis, n = 10 mice per group. Magnification bar = 170 µm. (B) MDA-231 or S1 cells were treated with AREG ligand with or without PAR34 antibody, and compared to PAR34 inhibition with EGF ligand treatment. Cell lysates were resolved on 8% SDS-PAGE gels before membrane transfer and probed with the corresponding tyrosine phosphorylated antibodies. (TIF) [file pone.0030255.s002.tif]

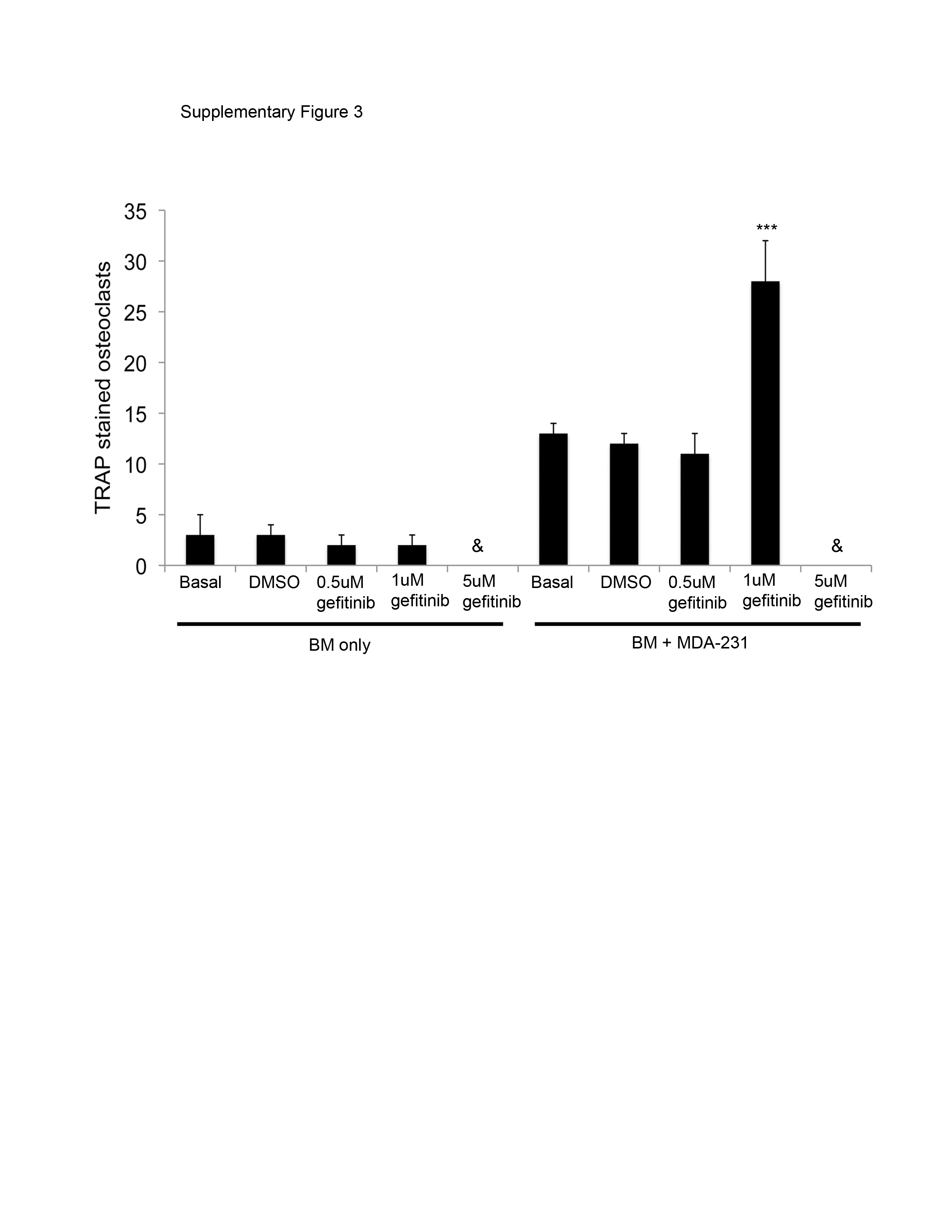

Supplement: Figure S3 — Gefitinib treatment of bone marrow co-cultures. Mouse bone marrow (BM) was cultured alone or co-cultured with MDA-231 cells followed by treatment with 0.5 µM, 1.0 µM, or 5 µM of EGFR kinase inhibitor gefitinib for three days. Osteoclasts were counted after TRAP staining from three random fields from two separate wells. *** p<0.001. & denotes all cultured cells in wells were dead after 5 µM gefitinib treatment. (TIF) [file pone.0030255.s003.tif]

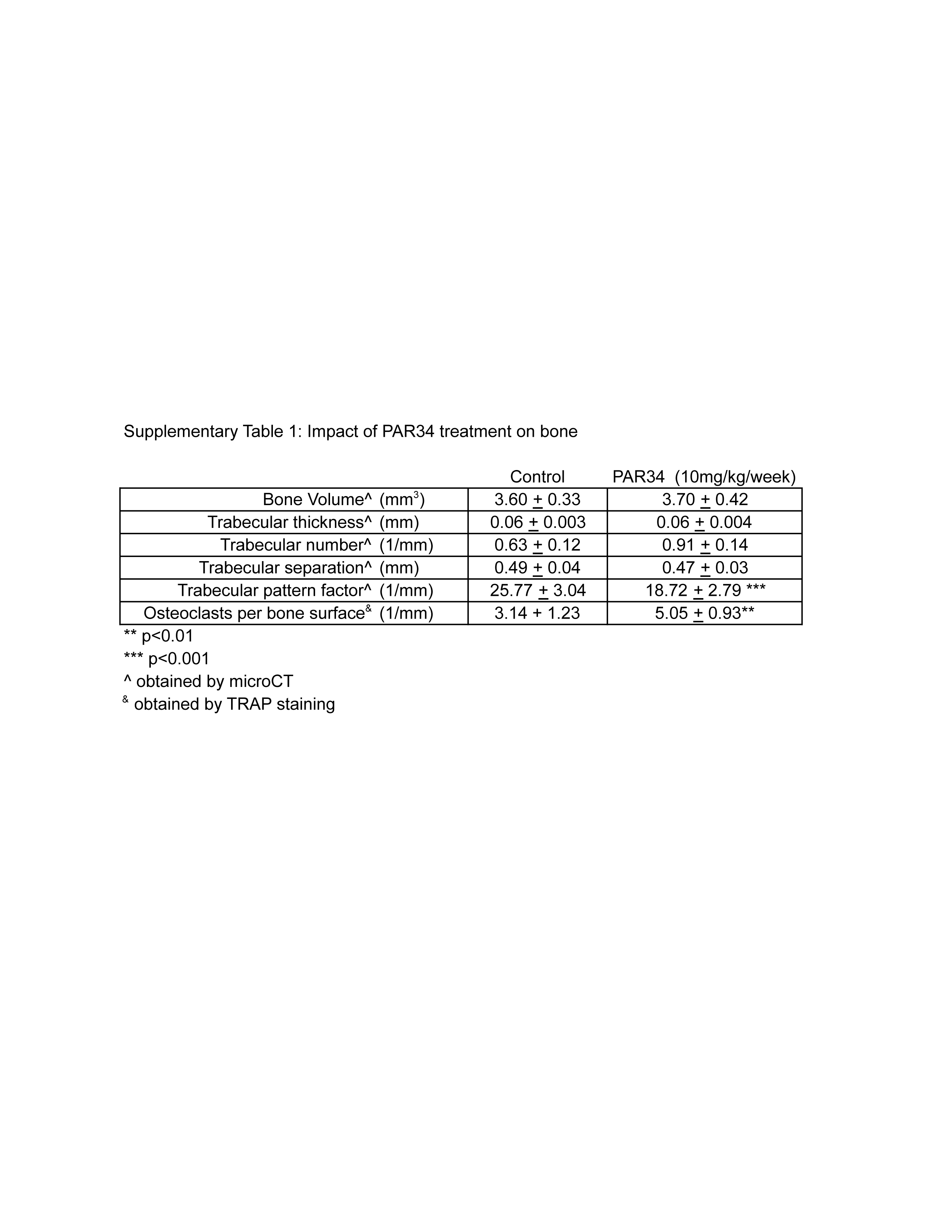

Supplement: Table S1 — MicroCT and histomorphometry measurements of PAR34 treated, non-tumor bearing tibiae. (TIF) [file pone.0030255.s004.tif]
