# Supplementary material for: Decreased Autocrine EGFR Signaling in Metastatic Breast Cancer Cells Inhibits Tumor Growth in Bone and Mammary Fat Pad
Source: PLoS One. 2012 Jan 19;7(1):e30255. doi: 10.1371/journal.pone.0030255 (PMC3261896; doi:10.1371/journal.pone.0030255)
Supplement: Methods S1 — Supporting Materials and Methods. (DOC) [file pone.0030255.s005.doc]

Materials and Methods S1

*Pharmacologic reagents*

Mouse EGF was purchased from Sigma. Recombinant human ligands for TGFα, HB-EGF, EGF, and AREG, as well as Normal Goat IgG, were purchased from R&D Systems (Minneapolis, MN). PAR34 clone was a gift from PDL BioPharma, and was harvested from mouse hybridomas, purified over a protein A column, and dialyzed into sterile 0.9% saline for injection. PD153035 was purchased from Tocris (United Kingdom). VEGF antibody was purchased from AbCam (Cambridge, MA). MMP-9 anitbody was purchased from Santa Cruz Biotechnology. PTHrP (1-34) ligand was purchased from Bachem. EGFR, ErbB2, ErbB3, and Erbb4 antibodies were purchased from Santa Cruz Biotechnology. Gefitinib was purchased from Tocris Bioscience (Ellisville, MO).

*Ligand and MCSF-1 ELISA assays*

MDA-231 cells were grown to confluence in a 12-well dish and serum starved overnight. Conditioned media was collected and cleared by centrifugation for 10 minutes at 4°C. To measure concentration of ligand attached to cell membrane, cells were harvested in ligand extract solution (1M tris-HCl, 0.5M EDTA, 10% TritonX-100, protease/phosphatase cocktail) and cleared through centrifugtion for 10 minutes at 4°C. Ligand concentrations (EGF, AREG, HB-EGF, Betacellulin, TGFα) were measured using the manufacturer’s instructions for the respective ligand DuoSet ELISA kit (R&D Systems). For MCSF-1 ELISAs, cells were treated for 6 hours with PD153035 or 24 hours with PAR34. After various time points, media was harvested from the wells and assayed per the manufacturer’s instructions. Samples were assayed in triplicate.

*Analyses of EGFR phosphorylation and expression*

We adapted previously published procedures for assaying EGFR ligand stimulation by assessing tyrosine phosphorylation [1]. Briefly, cells were plated on a 100mm dish and grown to confluence. Cells were incubated on ice for 30 minutes, washed with ice cold PBS and treated with ligand for 7 minutes. Cells were lysed and incubated on ice for 20 minutes. Lysates were cleared by centrifugation at 13k rpm for 10 minutes at 4°C, and transferred to a fresh tube.

Concanavalin A-sepharose beads were used to precipitate glycoproteins (which include ErbB receptors) from cleared cell lysates. Precipitates were resolved by SDS-PAGE on a 7.5 % polyacrylamide gel and electrotransferred to PVDF membrane (BioRad). Blots were probed using an anti-phosphotyrosine mouse monoclonal antibody (Upstate Biotechnology) or an anti-phosphospecific rabbit monoclonal antibodies (Cell Signaling Technology). Primary antibody binding was detected using a goat anti-mouse or goat anti-rabbit antibody conjugated to horseradish peroxidase (Kirkegarrd and Perry Labs Inc) and enhanced chemiluminescence (Santa Cruz). Immunoblots were then stripped and reprobed with an anti-EGFR rabbit polyclonal antibody (Santa Cruz) and detected as described above.

*RNA isolation and quantitative real-time reverse transcription PCR (Q-RT-PCR)*

Total RNA was prepared using the mini RNA isolation II kit from Zymo Research Corporation according to the manufacture’s instructions. Reverse transcription (RT) and quantitative real-time PCR (QRT-PCR) was performed as previously described [2]. Quantitative real-time PCR (QRT-PCR) was performed using DyNAmo HS SYBR Green qPCR master mix (New England Biolabs) according to the manufacturer’s instruction. PCR reactions were performed in a DNA Engine Opticon System (MJ Research Inc), primers and temperatures are listed in [3].

*Immunohistochemistry*

Histological tumor sections were rehydrated through alcohol gradation, followed by antigen retrieval for 20 minutes in boiling Tris/EDTA pH 9.0. Sections were blocked in 10% serum for 2 hours, followed by primary antibody for CD31 or Ki67 at 4°C for 24 hours. Sections were then incubated in peroxidase blocking solutions (3% H_2_O_2_ in TBS) for 15 minutes. Secondary biotinylated-antibody was added for 1 hour at room temperature (Vector Laboratories). Primary antibody was detected using HRP-Streptavidin and DAB peroxidase (both Vector Laboratories). CD31 stained sections were counterstained with hematoxylin, Ki67 slides were not counterstained, and all slides were dehydrated and cleared through xylene before mounting.

*Histomorphometry*

Bones: Histomorphometry was performed using a Leica DM2500 microscope, fitted with Q-imaging Micropublisher Camera (W. Nuhsbaum Inc., McHenry, IL). Histomorphometric analysis was performed with Bioquant OsteoII 2010 software (Bioquant image analysis corporation, Nashville, TN). Tumor volume was measured on H&E stained bone sections. Osteoclasts that were at the tumor-bone interface were counted at 20X on the tumor-bearing bones, and reported as osteoclasts per tumor bone interface. Osteoclasts in non-injected mice given therapeutics only, were counted in the trabecular region directly underneath the growth plate and reported as osteoclasts per bone surface [4,5]. Care was taken in measuring the same size region for each bone.

Tumors: Ki67 tumor counts: Four 10X images were randomly captured from the cortex of each tumor avoiding the necrotic centers. From each of these images all the labeled cells in 0.1 mm^2^ area were counted and a mean number of positive cells for each tumor generated. Counts from each of the shEGFR-MDA-231, shControl, and PAR-34 treated tumors were averaged. Differences in counts among the tumors were not significant. Necrosis: 10X images were obtained for each H&E stained tumor. Using ImageJ (NIH) the entire tumor area was measured and compared to only the necrotic areas of each tumor. Percent necrosis was calculated.

*MTT Assay*

500 cells per well were plated in a 96-well dish, in quadruplicate wells per cell line, and allowed to sit overnight in a 37°C/5% CO_2_ incubator. PAR34 or control IgG was plated at 10μg/mL when specified. MTT measurements were taken on days 1, 3, 5, and 7 after plating. MTT working solution was used at 1mg/mL MTT in cell culture medium. On days of measurement, 50μL of MTT working solution was added to each well and incubated for 4 hours at 37°C/5% CO_2_. Medium was removed and 150μL DMSO added to each well and read on a 96-well plate reader at 600nM.

*Migration Assays*

BD BioCoat™ Control Inserts were purchased from Becton Dickinson, and used per manufacturers instructions. For each insert, 1x10^5^ cells per well were plated and allowed to migrate or invade for 24 hours as in [6]. After 24 hours, each insert was fixed and stained using the Hema3 Stat Pack (Fisher Scientific) per manufacturer’s instructions, and allowed to dry overnight. The next day, each insert was carefully cut from the plastic insert using a scalpel blade, and placed on clean microscope slides sealing with microscope oil. Assays were performed in duplicate migration chambers, with four random images taken per chamber.

**References**

1. Gilmore JL, Riese DJ, 2nd (2004) secErbB4-26/549 antagonizes ligand-induced ErbB4 tyrosine phosphorylation. Oncol Res 14: 589-602.

2. Gilmore JL, Scott JA, Bouizar Z, Robling A, Pitfield SE, et al. (2008) Amphiregulin-EGFR signaling regulates PTHrP gene expression in breast cancer cells. Breast Cancer Res Treat 110: 493-505.

3. Cho YM, Lewis DA, Koltz PF, Richard V, Gocken TA, et al. (2004) Regulation of parathyroid hormone-related protein gene expression by epidermal growth factor-family ligands in primary human keratinocytes. J Endocrinol 181: 179-190.

4. Dunn LK, Mohammad KS, Fournier PG, McKenna CR, Davis HW, et al. (2009) Hypoxia and TGF-beta drive breast cancer bone metastases through parallel signaling pathways in tumor cells and the bone microenvironment. PLoS ONE 4: e6896.

5. Mohammad KS, Javelaud D, Fournier PG, Niewolna M, McKenna CR, et al. TGF-beta-RI kinase inhibitor SD-208 reduces the development and progression of melanoma bone metastases. Cancer Res 71: 175-184.

6. Kwon YJ, Hurst DR, Steg AD, Yuan K, Vaidya KS, et al. Gli1 enhances migration and invasion via up-regulation of MMP-11 and promotes metastasis in ERalpha negative breast cancer cell lines. Clin Exp Metastasis 28: 437-449.
